# Mediator kinase inhibition drives myometrial stem cell differentiation and the uterine fibroid phenotype through super-enhancer reprogramming

**DOI:** 10.21203/rs.3.rs-5125876/v1

**Published:** 2024-12-16

**Authors:** Subash Khadka, Brandon Lukas, Claire Xin Sun, Sribalasubashini Muralimanoharan, Karthigayan Shanmugasundaram, Azad Khosh, Claire Schenken, Nicholas Stansbury, Robert Schenken, Ron Firestein, Yang Dai, Thomas Boyer

**Affiliations:** UT Health San Antonio: The University of Texas Health Science Center at San Antonio; University of Illinois Chicago; Hudson Institute of Medical Research; UT Health San Antonio: The University of Texas Health Science Center at San Antonio; UT Health San Antonio: The University of Texas Health Science Center at San Antonio; UT Health San Antonio: The University of Texas Health Science Center at San Antonio; UT Health San Antonio: The University of Texas Health Science Center at San Antonio; UT Health San Antonio: The University of Texas Health Science Center at San Antonio; UT Health San Antonio: The University of Texas Health Science Center at San Antonio; Hudson Institute of Medical Research; University of Illinois Chicago; UT Health San Antonio: The University of Texas Health Science Center at San Antonio

## Abstract

Uterine fibroids (UFs) are the most common non-cutaneous tumors in women worldwide. UFs arise from genetic alterations in myometrial stem cells (MM SCs) that trigger their transformation into tumor initiating cells (UF SCs). Mutations in the RNA polymerase II Mediator subunit MED12 are dominant drivers of UFs, accounting for 70% of these clinically significant lesions. Biochemically, UF driver mutations in MED12 disrupt CDK8/19 kinase activity in Mediator, but how Mediator kinase disruption triggers MM SC transformation remains unknown. Here, we show that pharmacologic inhibition of CDK8/19 in MM SCs removes a barrier to myogenic differentiation down an altered pathway characterized by molecular phenotypes characteristic of UFs, including oncogenic growth and extracellular matrix (ECM) production. These perturbations appear to be induced by transcriptomic changes, arising in part through epigenomic alteration and super-enhancer reprogramming, that broadly recapitulate those found in MED12-mutant UFs. Altogether these findings provide new insights concerning the biological role of CDK8/19 in MM SC biology and UF formation.

## INTRODUCTION

Uterine Fibroids (UFs) or uterine leiomyomas are benign monoclonal neoplasms of myometrium and represent the most common non-cutaneous tumors in women worldwide [1]. Tumors occur in approximately 77% of women overall and are clinically manifest in roughly 25% by age 45 [2]. Although benign, these tumors are nonetheless associated with significant loss of quality of life and fertility in women through symptoms ranging from menorrhagia and pelvic pain to defective implantation, recurrent miscarriage, and preterm/obstructed labor [2, 3]. Unfortunately, current treatment options for UFs are limited. Curative surgery (hysterectomy) destroys reproductive function, while fertility-sparing surgical and radiologic alternatives are variously associated with reduced long-term reproductive function and/or high tumor recurrence rates [4, 5]. On the other hand, current hormonal therapies are contraindicated in women actively pursuing a pregnancy, and otherwise effective only during use, which is limited due to long-term safety and other concerns [4–6]. Accordingly, no long-term uterine-sparing treatment options exist for UFs, and better mechanistic insight into tumor etiology is key to develop newer targeted therapies.

The prevailing model for UF pathogenesis invokes a genetic transformation of a single myometrial stem cell (MM SC) into a tumor initiating stem cell (UF SC) that initiates and maintains monoclonal tumor growth characterized by cell proliferation and extracellular matrix (ECM) production under the influence of endocrine, autocrine and paracrine growth factor and hormone receptor signaling [3, 7]. Among the genetic drivers responsible for MM SC transformation, recurrent somatic mutations in the RNA polymerase II (RNAPII) transcriptional Mediator subunit MED12 are by far the most prevalent, accounting for more than 70% of UFs [8, 9]. The remaining tumor fraction comprises a heterogenous group characterized by genetic alterations leading to overexpression of HMGA1/HMGA2, inactivation of the SRCAP histone loading complex, or biallelic loss of fumarate hydratase [9, 10]. Regarding *MED12* alterations, all reside in exons 1 or 2 and most are missense mutations, with a minor proportion corresponding to small insertion/deletions that nonetheless retain the *MED12* open reading frame [8, 11]. Along with their high frequency occurrence, two additional findings suggest that MED12 mutations are tumor drivers. First, predominant monoallelic expression of mutant *MED12* is universally observed in human UFs, indicating selection for a functionally altered allele during tumorigenesis [8, 11]. Second, expression of an orthologous UF-linked *Med12* mutant transgene in mice elicits tumor formation, providing direct genetic proof of disease causality [12].

MED12, along with MED13, CCNC, and CDK8 (or its paralog CDK19) comprise a 4-subunit ‘kinase’ module (MKM) that associates with a 26-subunit Mediator core [13]. The intact 30-subunit Mediator is an evolutionary conserved multiprotein assembly that bridges enhancer-bound and signal activated transcription factors to RNA polymerase II, thus serving to channel regulatory signals from activator and repressor proteins to affect changes in gene expression programs that control diverse physiological processes, including cell growth and homeostasis, development, and differentiation [14]. CDK8/19, the only catalytic subunits within Mediator, are atypical CDKs that lack an otherwise conserved threonine residue in their respective activation (T)-loops known to be targeted in most CDKs for activating phosphorylation by the CDK-activating kinase (CAK) [15]. Accordingly, CDK8/19 are activated non-canonically in a manner that we and others showed instead requires MED12 [16–18]. First, we found that MED12 activates CycC-CDK8/19 by binding directly to both dimeric CycC-CDK subunits and, further, that all UF driver mutations in MED12 lie within its CycC-CDK8/19 binding and activation domain [19, 20]. Second, we confirmed that all UF driver mutations in MED12 disrupt CycC-CDK8/19 activation in vitro and, importantly, in clinically relevant patient fibroids [16, 17]. More recently, Cryo-EM structures of both the yeast and human Mediator kinase module confirmed these biochemical findings and revealed that UF driver mutations in MED12 disrupt its ability to bind and stabilize the CDK8 T-loop, leading to impaired kinase activity [13, 21]. Collectively, these findings reveal the first and heretofore only known biochemical defect arising from UF driver mutations in MED12, and further imply an etiological role for CDK8 in UF pathogenesis. However, the mechanistic basis by which CDK8 dysfunction drives the development of MED12-mutant UFs, the predominant UF subclass, remains to be established.

Consistent with the stem cell (SC) model for UF pathogenesis, stem-like cells have been isolated using a variety of approaches from both myometrium (MM) and UF tumors [22–26]. These approaches have ranged from those dependent on the unique properties of SCs [23, 25] to those based on the presence of mesenchymal progenitor cell-related markers (Stro-1^+^/CD44^+^ [22], CD34^+^/CD49b^+^ [24], CD34^+^/CD49f^+^ [26], SUSD^+^ [27]). Regardless of the approach used, the potential of these isolated SCs to undergo multilineage differentiation in vitro and initiate tumor formation in vivo has been established, supporting their true SC nature. Thus, while the presence of stem cells in both normal (MM) and diseased (UF) compartments supports the stem cell theory, the molecular basis by which a MM SC undergoes genetic transformation by a UF driver gene mutation into a tumor initiating UF SC is completely unknown.

To determine whether and how Mediator kinase disruption due to pathogenic mutations in MED12 programs MM SCs to acquire the UF phenotype, we profiled the transcriptomic and epigenomic changes induced by pharmacologic inhibition of CDK8/19 in patient-derived MM SCs. This strategy provided us a highly effective and experimentally flexible approach to model the immediate downstream biochemical defect arising from UF driver mutations in MED12 (disruption of Mediator kinase activity), permitting us to assess in real-time the earliest molecular changes arising from these pathogenic mutations.

## RESULTS

### Mediator kinase inhibition in MM SCs induces spontaneous myogenesis and triggers upregulation of oncogenic cell growth and ECM related pathways.

To explore the early molecular changes arising in MM SCs upon disruption of Mediator kinase activity, we first isolated resident MM stem/progenitor cells (MM SCs) from myometrial tissue of a 37 year-old Hispanic patient undergoing hysterectomy for reasons other than symptomatic UFs using a flow-cytometry based side population technique [25, 28] (Fig. S1A and B). We previously validated the mesenchymal origin (CD90+) of these MM side population cells [28]. Further, their undifferentiated status was confirmed by elevated expression of stem cell markers OCT4, NANOG, SOX2, SUSD2, and the ATP-binding cassette (ABC) transporter protein family member G2 (ABCG2), as well as low levels of nuclear hormone receptors and smooth muscle cell markers, relative to non-SP cells (Fig. S1C). As expected, Sanger sequencing confirmed that these MM SCs carry only WT *MED12* (data not shown).

To recapitulate the immediate downstream biochemical defect arising from UF driver mutations in MED12, we employed a highly specific and potent pharmacological inhibitor of CDK8/19 (CCT251545) [29, 30]. Target engagement and Mediator kinase inhibition by CCT251545 in MM SCs was confirmed by analysis of INFγ-stimulated STAT1 phosphorylation on serine residue 727 (pSTAT1^SER727^), a validated biomarker of Mediator kinase activity in vivo [28, 30]. Dose-response experiments revealed that maximal inhibition of pSTAT1^SER727^ was achieved with 40 nM CCT251545 with sustained inhibition for at least 24 hours in MM SCs (Fig. S2A and B), which is consistent with prior findings in COLO205 and SW620 colorectal cancer cell lines wherein 87.5 nM CCT251545 was required for maximal sustained inhibition of pSTAT1^SER727^ [30]. Because of possible variations is compound delivery and/or biological response, we elected to use 100 nM of CCT251545 for our experiments, which we confirmed to be sufficiently potent for Mediator kinase inhibition (Fig. S2C) with no noticeable effect on cell health which was monitored by microscopic visualization, cell counting, and molecular analyses following RNA-seq (see below).

**Figure 1:**
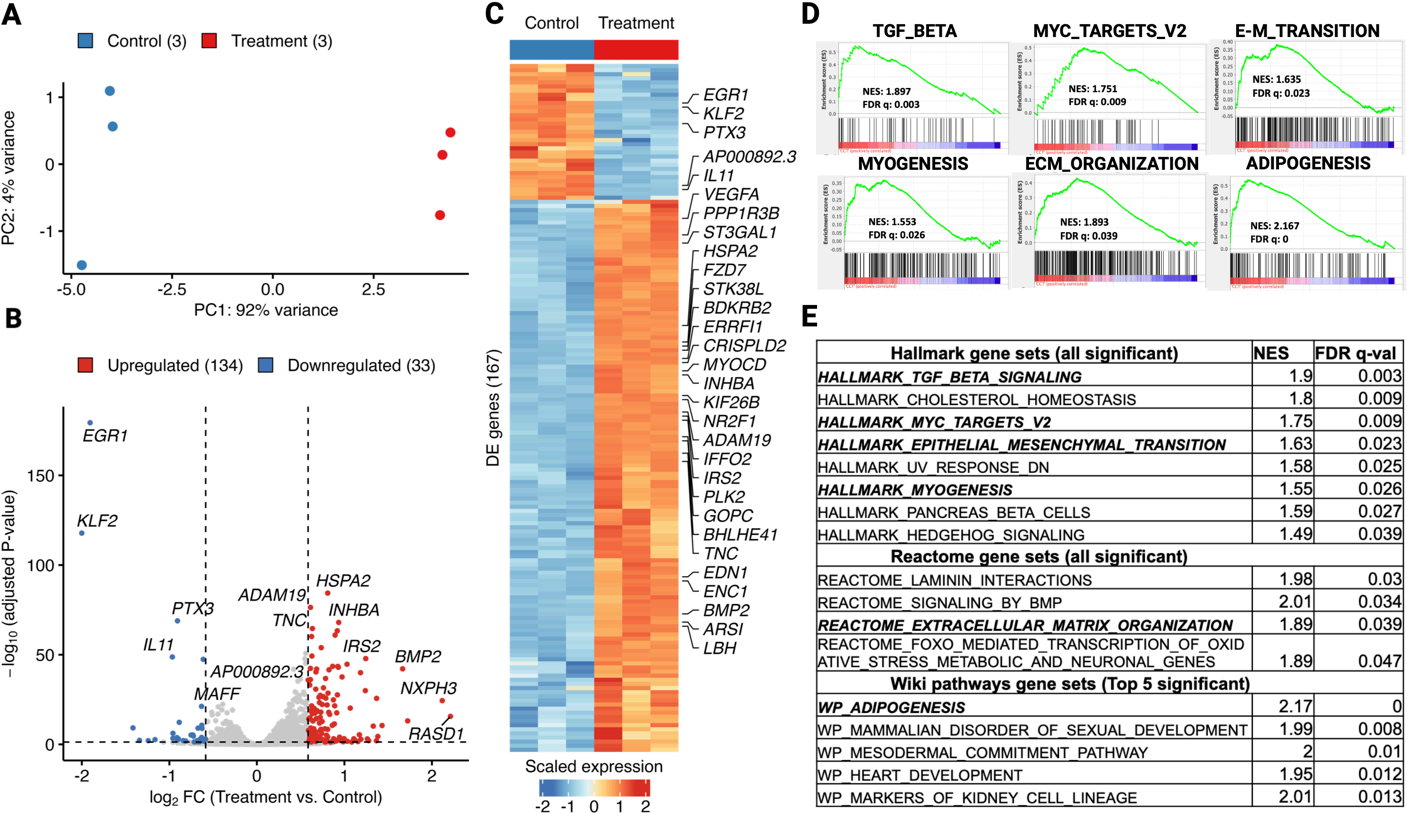
Short-term (4.5hr) Mediator kinase inhibition in MM SCs activates multilineage gene expression programs. **A.** Principal component analysis (PCA) plot of top 500 most variable genes in three replicate cultures of MM SCs treated with control DMSO (blue dots) or CCT251545 (red dots). **B.** Volcano plot showing 167 differentially expressed genes (DEGs; >1.5 absolute fold-change; Padj <0.05) in CCT251545-treated vs control MM SCs. Genes upregulated (n=134) and downregulated (n=33) are indicated by red and blue dots, respectively. **C.**Heat map of 167 DEGs with top 30 DEGs (ranked by adjusted *P* value) labeled by gene name. **D.** GSEA analysis of representative gene sets upregulated in MM SCs by 4.5hr treatment with CCT251545 includes TGF-Beta signaling, Myc Targets V2, E-M Transition, and Myogenesis (all Hallmark), as well as ECM Organization (Reactome) and Adipogenesis (WikiPathways). **E.**List of all significant (FDR-q val. < 0.05) gene sets from Hallmark and Reactome and top 5 from Wiki Pathways databases ranked by FDR q-values. Selected gene sets shown in **D** as GESA plots are italicized and bold.

**Figure 2:**
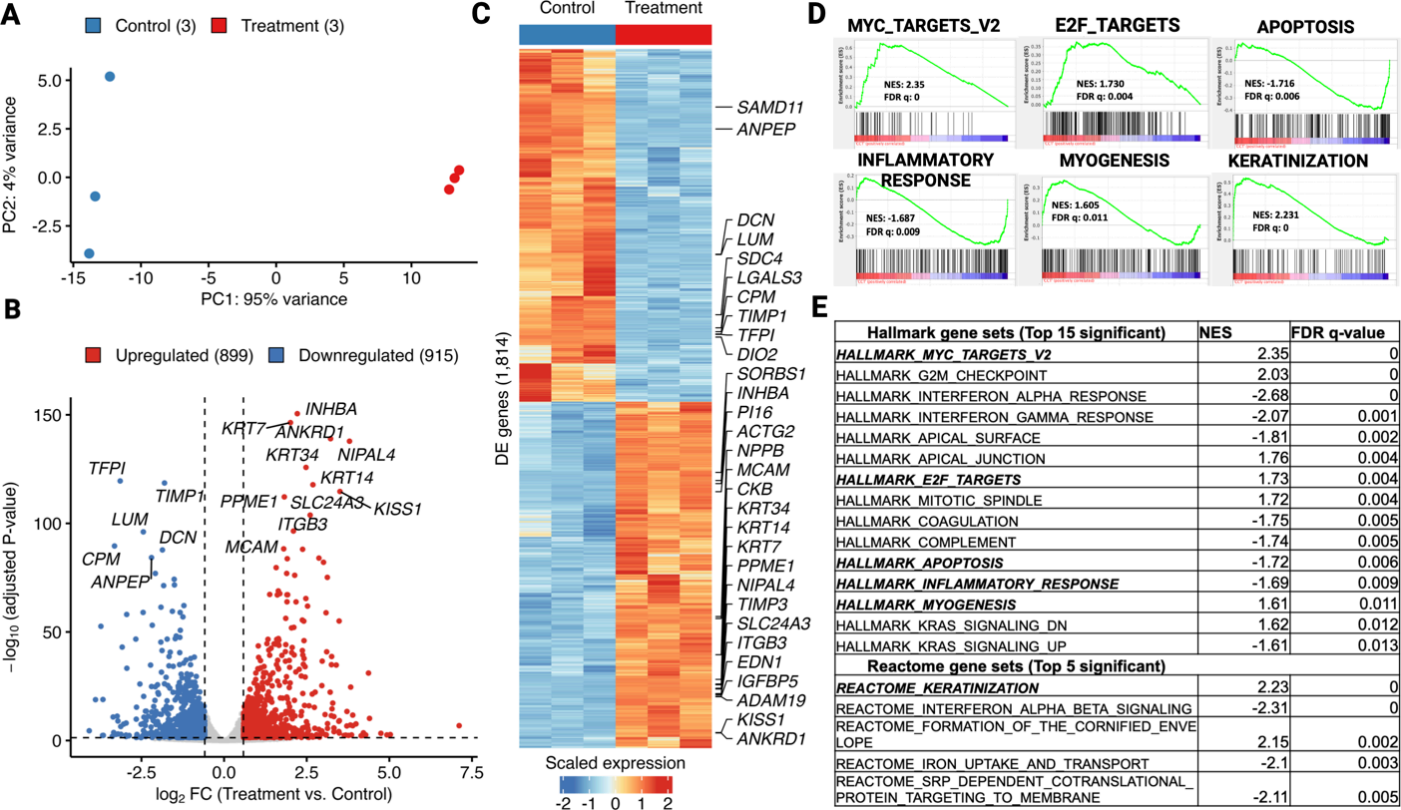
Long-term (3.5d) Mediator kinase inhibition in MM SCs broadly alters gene expression programs associated with myogenic differentiation and the UF phenotype. **A.** Principal component analysis (PCA) plot of top 500 most variable genes in three replicate cultures of MM SCs treated with control DMSO (blue dots) or CCT251545 (red dots). **B.**Volcano plot showing 1,814 DEGs (>1.5 absolute fold-change; Padj <0.05) in CCT251545-treated vs control MM SCs. Genes upregulated (n=899) and downregulated (n=915) are indicated by red and blue dots, respectively. **C.** Heat map of DEGs with top 30 DEGs (ranked by adjusted P value) labeled by gene name. **D.** GSEA analysis of representative gene sets upregulated in MM SCs by 3.5d treatment with CCT251545 includes MYC Targets V2, E2F Targets, and Myogenesis (all Hallmark), as well as Keratinization (Reactome). Representative gene sets downregulated include Apoptosis and Inflammatory Response (both Hallmark). **E.** List of all significant (FDR-q val. < 0.05) gene sets from Hallmark and top 5 from Reactome databases ranked by FDR q-values. Selected gene sets shown in **D** as GESA plots are italicized and bold.

Using this system, we first examined the transcriptomic changes induced by Mediator kinase inhibition in MM SCs. We used both short (4.5 hour) and long (3.5 day) treatment timepoints to capture direct and indirect effects on gene regulation, respectively. At both timepoints, PCA plots revealed clustering of samples by treatment groups, and separation of clusters along the X-axis (PC1) indicated that inhibitor treatment accounted for most (> 90%) of the variance in gene expression (Figs. 1A and 2A). Analysis of differentially expressed genes (DEGs; >1.5 absolute fold-change; Padj < 0.05) revealed that shorter treatment time induced fewer gene expression changes (167 total; 134 up and 33 down; Fig. 1B and C) than longer treatment time (1,814 total; 899 up and 915 down; Fig. 2B and C), although DEGs at both time points showed a high degree directionally concordant overlap (Fig. S3). These findings indicate that: (1) acute inhibition of Mediator kinase primarily activates gene expression and (2) the effects of Mediator kinase inhibition accumulate over time, leading to a more stabilized and balanced gene expression profile.

Gene set enrichment analysis (GSEA) revealed that Mediator kinase inhibition in MM SC cells led to upregulation of genes significantly linked to the Hallmark “Myogenesis” pathway (Fig. 1D and E; Fig. 2D and E), which was further supported by enrichment of terms associated with “Vascular Smooth Muscle Contraction” [KEGG] and “Smooth Muscle Contraction” [Reactome] in additional databases (Table S3). Notably, additional differentiation pathways, including “Adipogenesis”, and “Osteoblast Differentiation”, as well as cell plasticity-associated pathways, including “Embryonic Stem Cell Pluripotency” and “Mesodermal Commitment”, were all found significantly enriched in the Wiki Pathways database (Table S2). These findings suggest that Mediator kinase might function as a general barrier to MM SC differentiation, and is consistent with the established role of CDK8 in the regulation of tissue differentiation pathways [31–36].

In addition to myogenic differentiation pathways, GSEA also revealed that Mediator kinase inhibition is associated with upregulation of several Hallmark pathways linked to the UF phenotype, including aberrant cell growth and ECM remodeling. In this regard, UFs are characterized by aberrant growth reliant on both hormone-dependent and hormone-independent mechanisms. Concomitantly, like other fibrotic diseases from which they derive their eponymous name, UFs are characterized by excessive production and reorganization of the ECM driven in part by growth factors including the TGF-β superfamily. The relationship between cell proliferation and ECM deposition within UFs is complex and the two processes are likely linked, as ECM is known to function as a reservoir for growth factors, some of which also contribute to further ECM production that contributes to tumor mass [37]. We observed that short term (4.5 hour) inhibitor treatment in MM SCs led to upregulation of genes enriched for “MYC Targets” as well as “TGF-β Signaling” and “ECM Organization” (Fig. 1D and E), while longer-term (3.5 day) treatment induced genes associated with “MYC Targets” and “E2F Targets” (Fig. 2D and E). Analysis of additional databases reinforced enrichment results from these Hallmark gene sets. For example, ECM-related terms such as “TGFβ” (KEGG), and “ECM Organization”, “Laminin Interactions”, and “Non-Integrin Membrane-ECM Interactions” (Reactome) were found, while cell growth and cell-cycle related terms, including “Hedgehog Signaling” (KEGG and Hallmark), “G_2_/M Checkpoint”, “Mitotic Spindle” and “mTORC1 Signaling” (Hallmark) were also present (Tables S2 and S3).

On the other hand, genes downregulated by Mediator kinase inhibition (at 3.5d) were enriched for Hallmark pathways linked to “Apoptosis”, and inflammation-related functions, including “Inflammatory Response”, “Interferon-α response”, and “Interferon-γ response” (Fig. 2D and E; Table S3). Interestingly, Epithelial Mesenchymal Transition (EMT) was an additional negatively enriched Hallmark gene set at 3.5d post-inhibitor treatment and, concomitant with downregulation of EMT, pathways implicated in cells with epithelial polarity (i.e., “Keratinization” and “Formation of the Cornified Envelope” [Reactome]) were enriched in additional databases (Fig. 2D and Table S3). Finally, GSEA revealed no negatively enriched Hallmark gene sets following short term (4.5 hour) inhibitor treatment, consistent with the observation that the vast majority of differentially expressed genes (80%) at this timepoint were upregulated (Fig. 1B and C).

**Figure 3:**
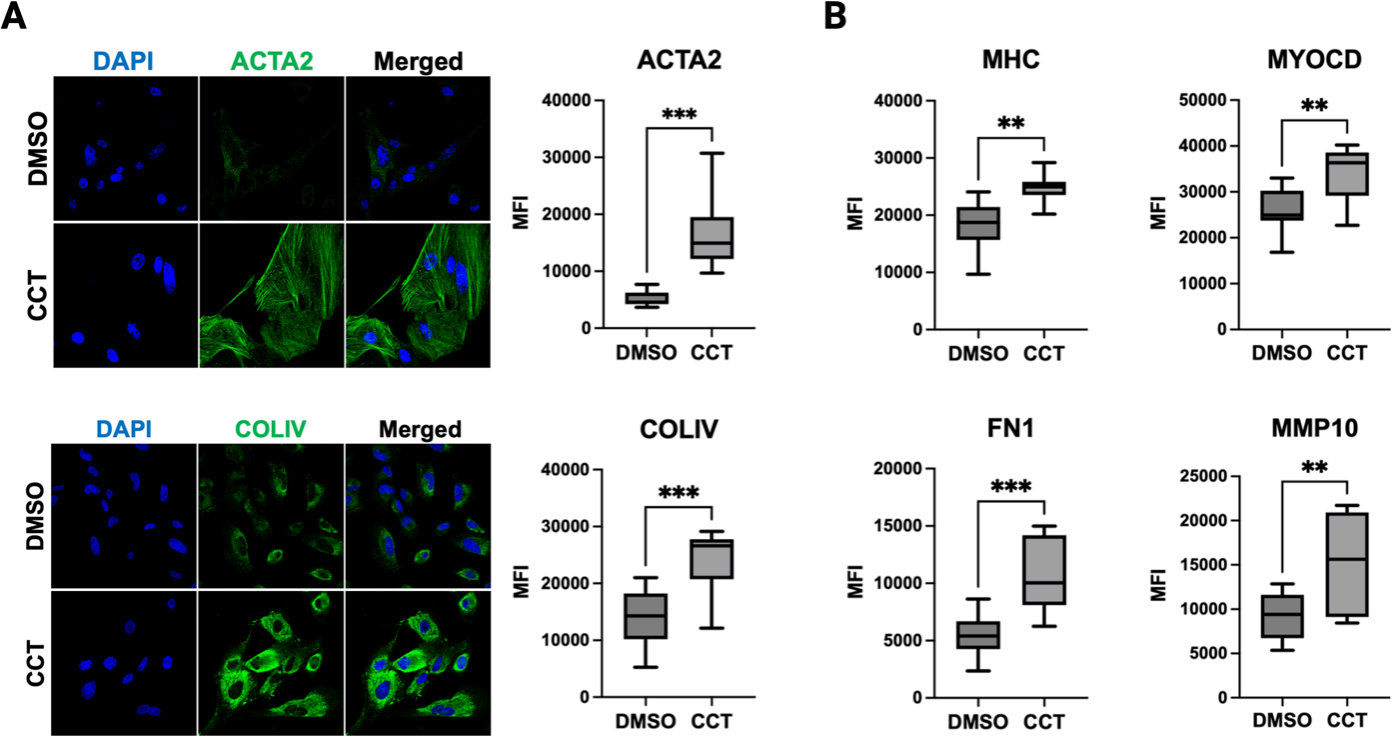
Mediator kinase inhibition upregulates markers of myogenesis and ECM remodeling. **A.** Representative immunocytochemical (ICC) staining and quantification for markers of myogenesis (ACTA2) and ECM (COL-IV) 3.5d post-treatment of MM SCs with DMSO or CCT251545 (CCT). **B.** Quantified ICC signals for additional markers of myogenesis (MHC, MYOCD) and ECM (FN1, MMP10) 3.5d post-treatment of MM SCs with DMSO or CCT251545 (CCT). Data represent quantified average fluorescent signals from 10 different fields in total from three independent experiments of control and CCT251545-treated cells. Images were taken in a single focal plane at 40X magnification using a ZEISS confocal imaging system. *P<0.05, **P≤0.01, ***P≤0.001; Unpaired t-test with Welch’s correction in GraphPad Prism10.

To validate findings from RNA-seq and subsequent GSEA analysis, we used immunocytochemistry to monitor the impact of Mediator kinase inhibition on expression of both myogenic and ECM markers in cultured MM SCs treated without or with CCT251545 for 3.5 days. We found that Mediator kinase inhibition induced markers of smooth muscle differentiation (smooth muscle actin, α2; Myosin Heavy Chain; and Myocardin) as well as ECM reorganization (Collagen Type IV, α1 chain; Fibronectin 1; and matrix metallopeptidase 10) (Fig. 3). Altogether, these findings suggest that Mediator kinase inhibition triggers spontaneous differentiation along an altered myogenic pathway characterized by phenotypes characteristic of UFs, including ECM and oncogenic growth.

### The aberrant transcriptional program induced by Mediator kinase inhibition in MM SCs is reminiscent of MED12-mutant uterine fibroid tumors.

**Figure 4:**
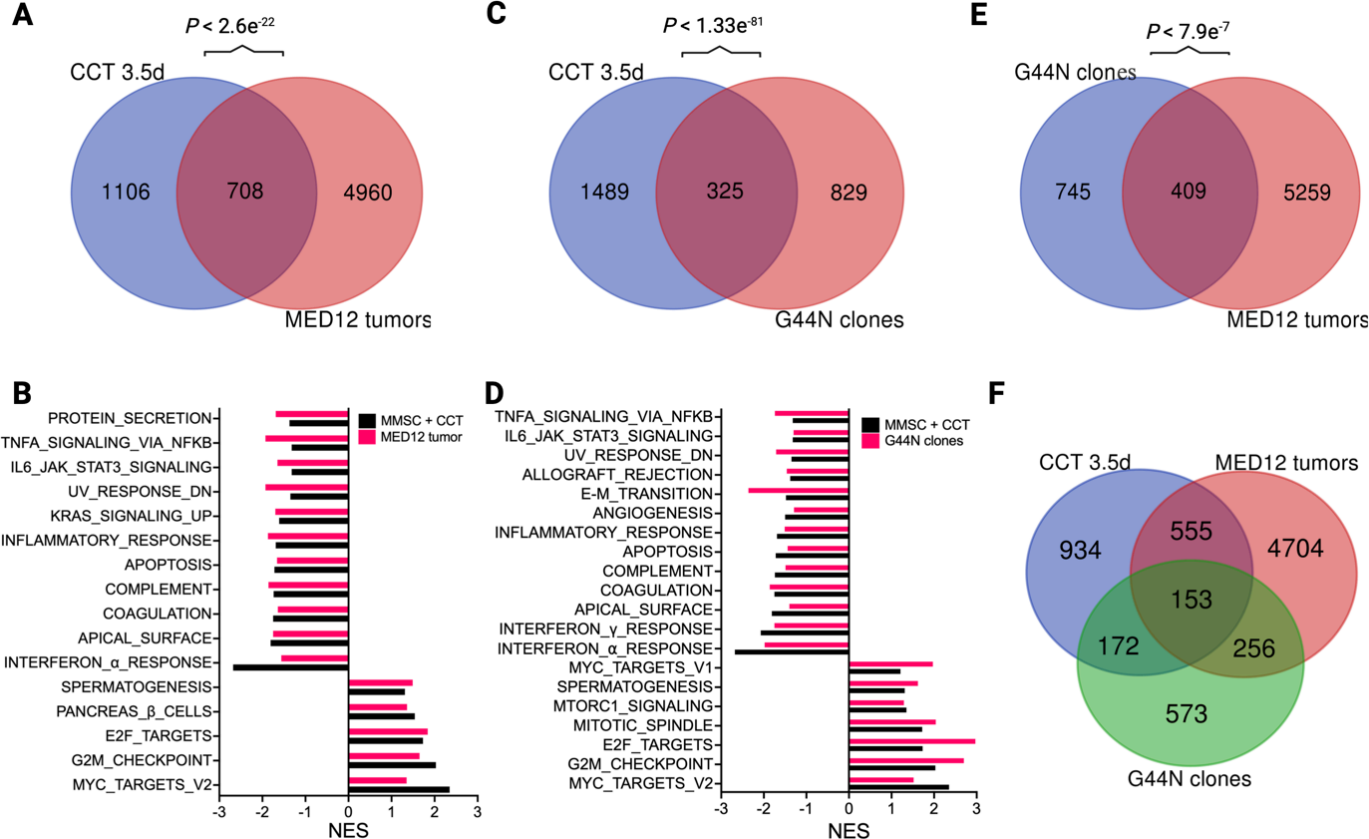
Mediator kinase inhibition in MM SCs induces a transcriptional program broadly reminiscent of MED12-mutant uterine fibroids. **A.** Venn diagram depicting overlap between DEGs in 3.5d CCT251545 (CCT)-treated MM SCs and MED12-mutant uterine fibroids [38]. Hypergeometric P value for the comparison of these two DEG sets is indicated above Venn diagram. **B.** Bar plot showing common Hallmark gene sets (significance threshold of 25% FDR) shared between CCT251545-treated MM SCs and MED12-mutant uterine fibroids. **C.** Venn diagram depicting overlap between DEGs in CCT251545-treated MM SCs and engineered G44N UtSMC clones [39]. Hypergeometric P value for the comparison of these two DEG sets is indicated above Venn diagram. **D.** Common Hallmark gene sets between CCT25154-treated MM SCs and engineered G44N UtSMC clones. **E.** Venn diagram depicting overlap between DEGs in engineered G44N UtSMC clones and MED12-mutant uterine fibroids. **F.** Venn diagram depicting 3-way overlap between DEGs in 3.5d CCT251545-treated MM SCs, MED12-mutant tumors, and G44N UtSMC clones.

Our observation that Mediator kinase inhibition induces molecular phenotypes in MM SCs characteristic of UFs prompted us to examine the degree to which DEGs (> 1.5 absolute fold-change; Padj < 0.05) in Mediator kinase-inhibited MM SCs are comparable and relevant to those found in UF tumors. To this end, we compared the 1,814 DEGs in MM SCs treated with CCT251545 for 3.5 days (Fig. 2C) with 5,668 DEGs found in patient-derived MED12-mutant UFs compared to their matched MM as reported previously by Moyo et al. [38]. Notably, 708 (~ 40%) were common between the two DEG sets, representing a significant overlap by hypergeometric testing (P < 2.6e^−22^; Fig. 4A). Furthermore, GSEA revealed multiple Hallmark pathways shared between Mediator kinase-inhibited MM SCs and MED12-mutant UFs (Fig. 4B). Up-regulated Hallmark pathways were highlighted by cell-cycle regulated gene sets, including “MYC Targets”, “E2F Targets”, and “G_2_/M Checkpoint” genes. Common down-regulated pathways included inflammatory and immune response gene sets as well as apoptosis.

We also compared the 1,814 DEGs in Mediator kinase-inhibited MM SCs to 1,154 DEGs (> 1.5 absolute fold-change; Padj < 0.05) identified through a comparative RNA-seq analysis of CRISPR-engineered G44N mutant MED12 uterine smooth muscle cells (UtSMCs) and their parental UtSMCs reported recently by Buyukcelebi et al [39]. Because > 50% of MED12 UF driver mutations in exon 2 correspond to missense mutations at Glycine 44, which we showed occupies a key role in CDK8 T-loop stabilization [13, 40], CRISPR-engineered G44N UtSMCs offer a viable genetic approach to model pathogenic MED12 mutations in a relevant myometrial smooth muscle cell line [39]. Notably, 325 genes were shared between the two DEG sets, representing a significant overlap by hypergeometric testing (P < 1.3e^−81^, Fig. 4C). Although DEGs in Mediator kinase-inhibited MM SCs overlapped to a greater extent with those in MED12-mutant G44N cells than they did with those in patient UFs (Fig. 4A vs 4C), they also showed more significant overlap with patient UFs than did MED12 G44N mutant cells (Fig. 4A vs 4E). Finally, we used principal component analysis to compare batch corrected transcriptomic profiles of all three data sets (MED12 mutant fibroids, engineered MED12 G44N UtSMCs, and CCT251545 treated MM SCs). This analysis revealed that PC1 segregates bulk tissues from cells (Fig. S4A) and, more importantly, MM SCs treated with CCT251545 for 3.5d were closer to MED12 mutant tumors than MED12 G44N UtSMCs (Fig. S4B). Altogether, these data indicate that pharmacologic inhibition of CDK8/19 in MM SCs effectively models gene expression changes in patient-derived MED12-mutant UFs, and further supports the notion that disruption of Mediator kinase activity contributes prominently to the molecular pathogenesis of MED12-mutant tumors.

### The aberrant transcriptional program induced by Mediator kinase inhibition in MM SCs arises through an altered epigenome.

**Figure 5:**
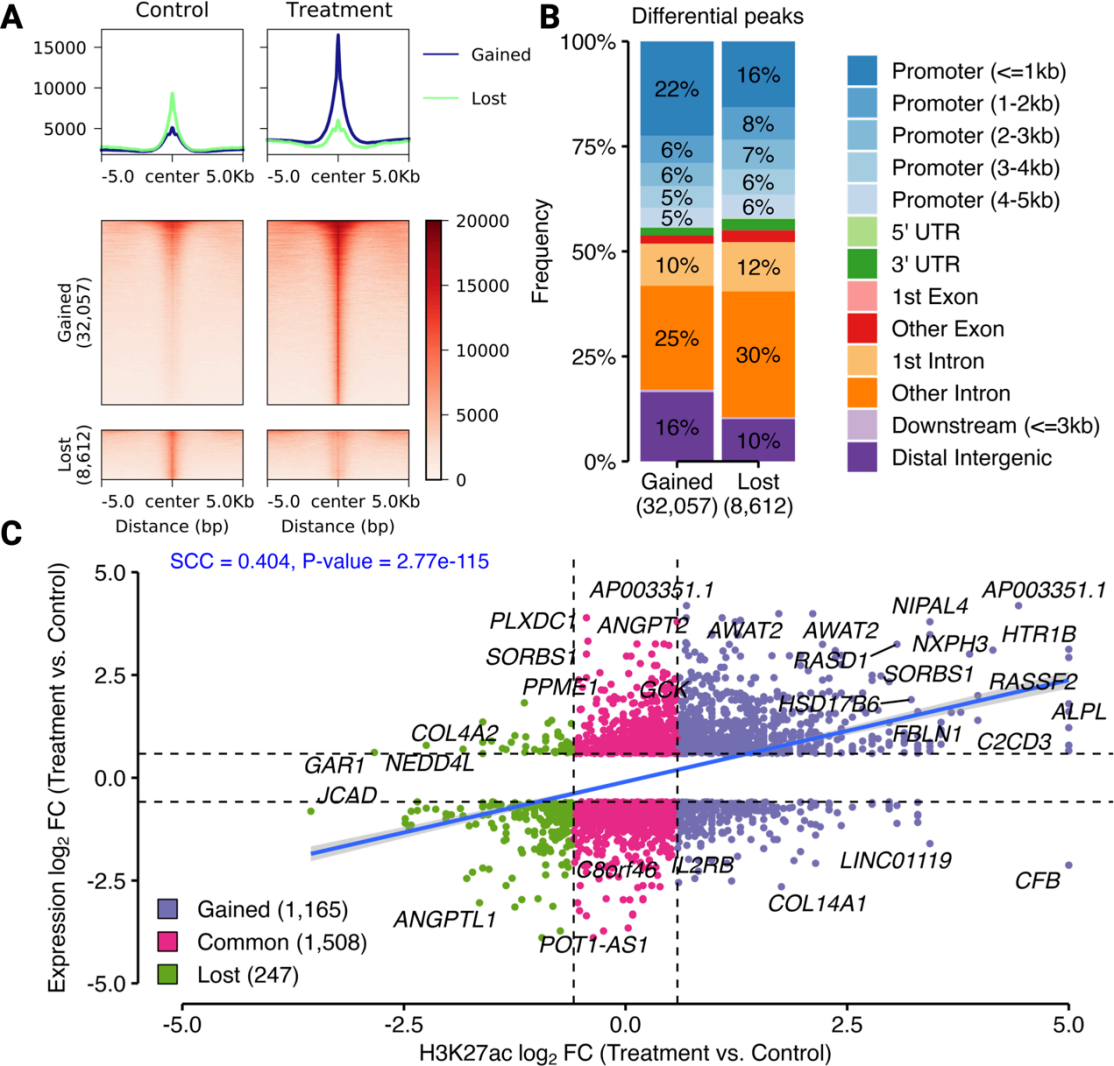
Mediator kinase inhibition in MM SCs reprograms the H3K27ac epigenome. **A.** Heat map of differential H3K27ac peaks in both control (DMSO) and 3.5d CCT251545 (treatment) conditions (one sample per condition) shown around 5kb from the peak center. The density plots above the heat maps demonstrate that the average intensity of gained peaks upon treatment was much higher than that of lost peaks. **B.** Bar plot showing genomic distribution of gained and lost H3K27ac peaks in 3.5d CCT251545-treated MM SCs. Overall, H3K27ac peaks on intronic regions were lost while those on proximal promoters and distal intergenic regions were gained upon inhibitor treatment. Note that panels **A** and **B** represent qualitative assessments to provide a visual comparison between conditions and no formal statistics were applied. **C.** Scatter plot of changes in expression and H3K27ac (log_2_FC each) occurring in MM SCs 3.5d post CCT251545-treatment vs control. H3K27ac changes include only those found within 5Kb of the TSS of DEGs. The numbers of H3K27ac peaks gained (violet dots), common (pink dots), and lost (green dots) between samples are indicated. The Spearmen correlation coefficient (SCC) is shown. Statistics applied in panel **C** are described in Experimental Procedures.

We sought to determine if the differential gene expression that we observed in Mediator kinase-inhibited compared to control MM SCs occurs through corresponding changes in the epigenome. Accordingly, we performed epigenomic analysis following long term (3.5 day) inhibitor treatment to monitor the cumulative effects of Mediator kinase inhibition when gene expression changes are stabilized and more effectively model patient UFs (Fig. S4B). To profile the epigenome, we used CUT&RUN to obtain genome-wide maps of Histone H3 Lysine 27 acetylation (H3K27ac), which marks transcriptionally active enhancers and promoters [41, 42]. Differential peak analysis identified 32,057 gained and 8,612 lost H3K27ac peaks in response to inhibitor treatment, indicating that Mediator kinase inhibition in MM SCs results in increased genomic activity at most regulatory elements (Fig. 5A). Moreover, the H3K27ac peaks gained by treatment belonged to proximal promoter and distal intergenic (enhancer) regions, suggesting that long-range genomic interactions were affected (Fig. 5B). Notably, correlation analysis of the combined H3K27ac and gene expression changes in MM SCs as a function of CCT251545 treatment revealed a positive association between H3K27ac peak strength and gene expression (Fig. 5C), indicating that gain and loss of H3K27ac is generally associated with enhanced and reduced gene expression, respectively. These results are consistent with the notion that gene expression changes arising in Mediator kinase inhibited MM SCs involve reprogramming of the H3K27ac epigenome.

### Mediator kinase inhibition in MM SCs leads to super-enhancer reprogramming as a basis for differential expression of cell identity genes.

**Figure 6:**
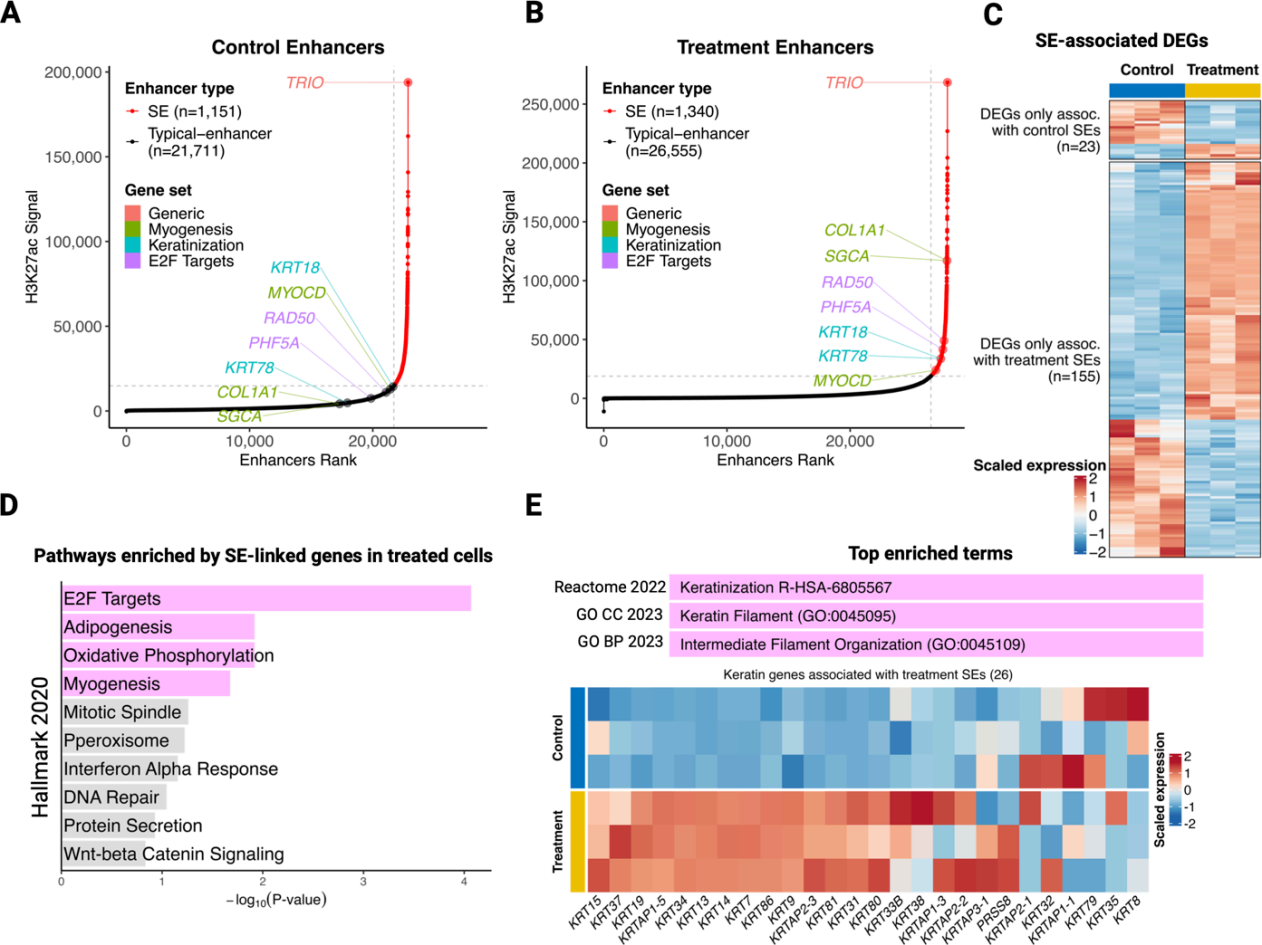
Mediator kinase inhibition in MM SCs activates key cell identity genes through SE acquisition. **A** and **B.** Rank Ordering of Super Enhancer (ROSE) plots show enhancer ranking based on H3K27ac signal intensities under control (DMSO) and CCT251545 (treatment) conditions. Representative genes that gained SEs upon CCT251545 treatment are highlighted in color and correspond to Hallmark gene sets as indicated (Myogenesis [olive], E2F Targets [purple] and keratinization [cyan]). **C.** Heat map of SE-associated DEGs shows overall upregulation of SE-linked genes in CCT251545-treated cells and the opposite trend in control (DMSO)-treated cells. **D.** Hallmark gene sets identified through functional enrichment (Enrichr) analysis of genes (1,712) associated with SEs in CCT251545-treated cells. The bar plot shows the top 10 enriched Hallmark gene sets ranked according to their Padj values. Pink bars correspond to significantly enriched gene sets (Padj <0.05), while gray bars correspond to gene sets that failed to reach significance. Note that significantly enriched Hallmark gene sets include those linked to cell growth (E2F Targets) and differentiation (Myogenesis, Adipogenesis). **E.** Enrichr analysis of 1,712 genes associated with SEs in CCT251545-treated cells also identified keratin related terms across multiple databases as the top enriched pathway (pink bar), which was further supported by upregulation of SE-linked keratin genes upon CCT251545 treatment (heat map).

Because MED12-dependent CDK8/19 activation regulates enhancer-promoter interactions and CDK8 appears to function as a barrier to cellular reprogramming [14, 43], we sought to determine if DEGs identified in Mediator kinase-inhibited MM SCs could be explained by altered enhancer activity. For this purpose, we chose to focus on super-enhancers (SEs), clustered enhancer elements responsible for control of key cell identity genes [44]. To identify SEs, we used our genome-wide maps of H3K27ac and the ROSE (Rank Ordering of Super-Enhancers) algorithm [44], which involved enhancer ranking based on H3K27ac signal intensity. Using ROSE, we identified 1,151 and 1,340 H3K27ac-defined SEs in control and Mediator-kinase inhibited MM SCs, respectively (Fig. 6A and 6B). This indicates an increased SE number upon CDK8/19 inhibition, which agrees with prior observations in acute myeloid leukemia (AML) cells and human and mouse embryonic stem cells [45, 46]. Consistent with these observations, we also found that the number of unique SE-associated genes was much higher in Mediator kinase-inhibited (1,712) compared to control (419) cells. Importantly, most of the DEGs associated with SEs in treated cells (155) were upregulated while those associated with SEs in control cells (23) were mainly downregulated (Fig. 6C). Furthermore, subsequent ‘Enrichr’ [47] analysis of the 1,712 genes associated with SEs only in treated cells revealed significant enrichment for Hallmark ‘E2F targets’ and ‘Myogenesis’ pathways, two of the prominently upregulated pathways at the RNA level in Mediator kinase-inhibited cells (Fig. 6D; Fig. 2D and E).

Interestingly, ‘Adipogenesis’, another enriched pathway among SE-linked genes in inhibitor-treated cells (Fig. 6D), was significantly enriched by GSEA at short-term (4.5h) treatment, although it did not reach significance at longer-term (3.5d) treatment (Fig. 1D and E; Table S2). Since we found that Mediator kinase inhibition is generally associated with gain of SEs, consistent with prior reports [45, 46], we focused on genes linked to SEs only in inhibitor-treated cells. Unexpectedly, Enrichr analysis using ‘Gene Ontology’ and ‘Reactome’ databases concordantly and most significantly enriched for ‘keratin’ related terms (Fig. 6E). Because keratins function prominently in epithelial cells, but have nonetheless been shown to be expressed in differentiating mesenchymal cells [48, 49], we further investigated and found that almost all SE-associated keratin genes gained expression upon Mediator kinase inhibition (Fig. 6E). This indicates that Mediator kinase inhibition in MM SCs induces a transient state of plasticity along the epithelial-mesenchymal continuum. Strikingly, and supporting our enrichment analysis of gained SE-linked genes, we were able to demonstrate the association of representative genes from Hallmark ‘Myogenesis’ (MYOCD, SGCA, COL1A1), ‘E2F targets’ (RAD50, PHF5A) and ‘Keratinization’ (KRT78, KRT18) pathways with SEs in treated cells, which were otherwise regulated by typical enhancers in control cells (Fig. 6A vs. 6B). Overall, these findings suggest that transcriptional alterations in Mediator kinase-inhibited MM SCs are regulated, at least in part, by altered SE engagement, and support the notion that key cell identity related pathways (such as ‘Myogenesis’ and ‘Keratinization’) are associated with SE acquisition.

## DISCUSSION

Although UFs are believed to arise clonally from dysregulated MM SCs and the vast majority (~ 80%) of these tumors harbor MED12 mutations, the basis by which these mutations drive tumorigenic transformation remains unclear. Because we previously showed that UF driver mutations in MED12 disrupt CDK8/19 kinase function [13, 16, 28], we profiled the transcriptome of CDK8/19-inhibited MM SCs and found that Mediator kinase inhibition triggered spontaneous myogenesis down an altered pathway characterized by molecular phenotypes characteristic of UFs, including oncogenic growth and extracellular matrix (ECM) production. This suggests that Mediator kinase is a general barrier to MM SC differentiation, consistent with its role in regulating tissue differentiation in other adult stem cell settings [32, 34]. We propose that during the normal process of smooth muscle cell differentiation, this CDK8-imposed barrier is removed in a tightly regulated manner by physiologic inductive signals, permitting programmed differentiation of MM SCs into uterine smooth muscle cells. However, spontaneous disruption of CDK8 (through chemical inhibition or somatic MED12 mutation) instead triggers unprogrammed differentiation and fate conversion as a consequence of enhanced cell plasticity, permitting MM SCs to acquire mixed features of the myogenic lineage and other mesenchymal-determined fates (Fig. S5). This model is also consistent with recent reports that CDK8 is a general molecular barrier to multiple cell reprogramming processes, and thus a target for inducing plasticity and cell fate conversion [43]. For example, inhibition of CDK8 in human and mouse primed pluripotent stem cells (corresponding to E6.5 post implantation embryo) was previously shown sufficient to convert them to their naïve state of pluripotency (corresponding to E4.5 pre-implantation epiblast) by activating pluripotency programs and suppressing developmental programs [45, 50]. Further, using a transcriptome-based chemical screening approach in mouse and human fibroblasts, CDK8 inhibition was shown to induce multilineage priming and subsequent transformation into hepatocytes and adipocytes, respectively, along with suppression of inflammatory gene expression [43]. Accordingly, CDK8 appears to function as a common barrier in multiple cell reprogramming systems, and its inhibition seems to induce a state of plasticity providing an opportunity for the most dominant alternate identity factors (myogenic in MM SCs or adipogenic in fibroblasts) to determine cell fate.

Our discovery that CDK8 inactivation triggers MM SC plasticity and fate conversion may be relevant to the pathogenic mechanism of UF genetic subtypes other than those carrying mutant MED12. For example, genomic rearrangements leading to overexpression of HMGA2 represent the second-most frequent driver alteration in UFs, accounting for ~ 10% of tumors [8, 9]. Recent work has shown that forced overexpression of HMGA2 in myometrial smooth muscle cells drives their de-differentiation into a more plastic phenotype, one that shares transcriptomic profiles and hallmark features, including ECM, with HMGA2-overexpressing UFs [51, 52]. Furthermore, mutations in individual genes encoding the SRCAP histone loading complex collectively represent the third most frequent driver change in UFs [9], and SRCAP mutations were shown to reduce deposition of the histone variant H2A.Z and trigger aberrant upregulation of bivalent embryonic stem cell genes, possibly accounting for enhanced cellular plasticity on the path to tumor development [9]. Accordingly, the ability of key driver mutations to incite a state of cellular plasticity through transcriptomic reprogramming may represent a conserved mechanism of UF pathogenesis.

Mechanistically, several studies have linked CDK8 inhibition to cell fate transformation through global enhancer and SE activation. For example, in human ESCs, Mediator kinase inhibition was shown to reprogram the expression of SE-dependent genes necessary to revert primed pluripotent cells to a naïve pluripotent state [45]. Our observation that Mediator kinase inhibition in MM SCs promotes myogenic differentiation accompanied by reprogramming and acquisition of SEs linked to muscle-determining TFs, including MYOCD, a master regulator of smooth muscle and cardiac muscle cell fate [53] is consistent with such a mechanism.

Because of their consonant relationship with cell identity, Mediator and SEs are also inextricably linked with oncogenic fate. Thus, in a Mediator kinase-dependent manner, tumor cells are known to acquire oncogenic SEs that drive cell transformation [54, 55]. Our finding that Mediator kinase inhibition in MM SCs promotes E2F pathway activation accompanied by SE acquisition at E2F target genes suggests a possible basis to explain, at least in part, aberrant growth characteristic of UF tumors. However, newly acquired oncogenic fate requires additional processes to support continued tumor maintenance. [54, 56]. In line with this, we also observed downregulation of “apoptosis” and upregulation of cell growth pathways such as “Myc Targets” in CDK8 inhibited MM SCs. Altogether, our data suggest that oncogenic cell growth in Mediator kinase-inhibited MM SCs appears to involve, at least in part, SE reprogramming leading to activation of pathways that recapitulate many of those found in patient-derived MED12-mutant tumors.

Given the mesenchymal origin of MM SCs, we were intrigued to observe SE-driven upregulation of keratin genes that function to maintain the mechanical integrity and stability of single epithelial cells and epithelial tissue through cell-cell contacts [57, 58]. In line with this, we found significant enrichment of GSEA terms in addition to “Keratinization”, including “Apical Junction”, “Adherens Junction Interactions”, and “Formation of the Cornified Envelope’ following long-term Mediator kinase inhibitor treatment (Table S3). Concordantly, we observed early (4.5hr) upregulation of EMT genes that was reversed by longer-term (3.5d) inhibiter treatment concomitant with enrichment of keratinization-related terms, possibly suggesting a switch in mesenchymal to epithelial polarity. While this phenomenon is in agreement with the notion that CDK8/19 inhibition relieves a barrier to cellular reprogramming, no studies have heretofore linked keratinization with UFs. Nonetheless, keratinizing cells are found in cervical carcinoma, and intriguingly, UFs have been reported to transcriptionally resemble cervical stroma more than the parent myometrium [59, 60]. Additional studies will be required to confirm and further explore this interesting observation.

Beyond SE reprogramming, the transcriptomic changes triggered by Mediator kinase-inhibition in MM SCs are likely to involve disruption of transcriptional programs with which CDK8 has been previously linked through SE-independent pathways. For example, CDK8 inhibition has been linked to upregulation of the TGF-β pathway, a known driver of ECM production, in a SE-independent manner [61]. Mechanistically, this occurs because TGFβ/BMP activated ‘SMADs’ require CDK8-dependent phosphorylation in the interdomain linker region for their ubiquitin-mediated turnover, which is blocked by CDK8 inhibition, leading to accumulation of activated SMADs. Notably, TGF-β-dependent accumulation of ECM in UFs can serve as the reservoir for profibrotic growth factors that cause further cell proliferation and ECM accumulation leading to growth of tumor volume [37]. Furthermore, CDK8 is known to suppress E2F1, releasing the block by E2F1 on β-catenin transcriptional activity, and subsequently activating WNT-signaling [62]. Thus, SE reprogramming may contribute significantly, but only partially, to the transcriptomic changes occurring in Mediator kinase inhibited MM SCs that drive the UF phenotype.

Finally, limitations of this study include the derivation of MM SCs from a single patient and use of a single sample for epigenetic profiling studies. Accordingly, patient heterogeneity and technical variability could combine to restrict the broader implications of this study. Nonetheless, our findings represent an important starting point for further investigation into the role of Mediator kinase as a barrier to cellular plasticity and fate conversion on the path to UF tumor development.

## EXPERIMENTAL PROCEDURES

### Isolation of MM SCs

Myometrial stem cells (MM SCs) were isolated from the non-diseased myometrium of an informed-consent 37-year old Hispanic female patient who underwent hysterectomy for reasons other than symptomatic UFs at the University of Texas Health Science Center at San Antonio (UT Health San Antonio). The UT Health San Antonio Institutional Review Board approved the protocol for recovery of surgical specimens. Patient-derived MM SCs were isolated using the using side population technique as previously described [25]. Briefly, hysterectomized myometrial tissue was collected in 1X Hank’s balanced salt solution (HBSS) (GIBCO) supplemented with 1X antibiotic-antimycotic (anti-anti, Thermo-Fisher Scientific) and chopped manually into pieces < 1mm^3^. Minced tissue was enzymatically digested in DMEM/F-12 medium (GIBCO) containing 10% FBS, 1% anti-anti, 10mM HEPES buffer (GIBCO), 2mg/ml type II collagenase (GIBCO) and 1mg/ml DNase I (Sigma-Aldrich) overnight (~ 16 hrs) at 37°C/150rpm. The dissociated single cell suspension was serially filtered through 100μm and then 40μm sterile cell strainers (BD-Falcon). The filtrate was washed with 1X HBSS, treated with erythrocyte lysis solution (ACK Lysis Buffer, GIBCO) for 5min at 4°C and washed again with 1X HBSS to remove the red blood cells.

The cells were resuspended at 2 × 10^^6^ cells/ml in 1X HBSS with 2% FBS and 10mM HEPES. Cells were then treated with 5μg/ml of fluorescent dye Hoechst-33342 (Sigma-Aldrich) in a 37°C water bath for 90 minutes. A small aliquot of cells that was treated with 50μm Reserpine (Sigma-Aldrcih) at 37°C/15mins prior to Hoechst-33342 treatment was used as negative control. After the incubation, cells were washed with 1X HBSS and resuspended back in 1X HBSS with 2% FBS and 1μg/ml Propidium Iodide (PI) (Sigma-Aldrich) prior to side population sorting in BD FACS ARIA II cell sorter. The collected side population cells (hereafter MM SCs) were subsequently cultured under hypoxic conditions (2% O_2_, 5% CO_2_) and subjected to Sanger sequencing to establish the MED12 mutation status before further analyses.

### MM SC culture and CCT251545 treatment

The MM SCs were cultured in 60mm dishes on Mesenchymal Stem Cell Growth Medium (MSCGM) BulletKit^™^ (PT-3001, Lonza) and incubated in hypoxia (2% O_2_, 5% CO_2_). Cells were ensured to be Mycoplasma free using MycoStrip test (InvivoGen). The MM SCs were then transferred from 60mm dishes to 10cm dishes and either split or frozen at ~ 80% confluency. Subsequently, the MM SCs were treated with DMSO or the CDK8/19-specific inhibitor CCT251545 (MedChemExpress) at 100nm for 4.5hrs and 3.5days. For 3.5day inhibitor treatment, fresh CCT251545 was replaced every 24 hours. A splitting ratio of 1 to 5 was used for cell passaging and CCT251545 treatment was initiated at passage 2 in 10cm dishes. One 10cm plate each of treated (CCT251545) or control (DMSO) cells were induced with 10ng/ml IFN-γ (Invitrogen) 45 minutes prior to harvesting to test STAT1-S727 phosphorylation levels using immunoblot as a readout of kinase inhibition by CCT251545. Remaining plates of inhibitor-treated or control cells were pooled, counted, and aliquoted for CUT&RUN reactions or individual plates harvested separately for RNA extraction.

### RNA-sequencing

Three independent replicates of MM SCs treated with CCT251545 or DMSO for 4.5h and 3.5d were used for RNA extraction using RNeasy mini kit (QIAGEN) and CCT251545 effectiveness in both treatment timepoints was validated by immunoblot for γ-interferon induced pSTAT1 S727 levels. RNA library preparation, quality control and sequencing were performed by Genome Sequencing Facility at UT Health San Antonio.

## CUT&RUN

CUT&RUN was performed using the protocol and reagents described before [63] with some modifications to adapt the workflow to perform in PCR tubes. Briefly, CCT251545 or DMSO-treated MM SCs (in parallel with those for RNA-sequencing) were pooled in 1.5ml EP tubes to have 500k cells per CUT&RUN reaction. Cells were washed twice at RT and resuspended in 200μl wash buffer/reaction before binding to activated Concanavalin-A coated magnetic beads for 10 mins at RT with rotation. Bead-bound cells were aliquoted into strip PCR tubes for each reaction/antibody (including the control IgG reaction) and were resuspended in 50μl antibody buffer (contains digitonin for permeabilization along with target antibodies) followed by overnight (~ 16hr) incubation at 4°C in a nutator. The cells were washed twice with cold digitonin buffer to remove excess and non-specifically bound antibodies, resuspended back in 50μl cold digitonin buffer, and treated with pAG-MNase (Epicypher) for 10min at RT in a nutator. Again, cells were washed twice with cold digitonin buffer, resuspended in 50μl cold digitonin buffer, and treated with 1μl of 100mM CaCl_2_ for 2 hours at 4°C to activate the MNase tethered to antibody-bound chromatin. MNase activity was stopped by adding 33μl of the 2X Stop buffer (containing 2pg/ml of *E. coli* spike-in DNA) to each tube and cleaved chromatin was released by incubating the tubes at 37°C in a thermocycler for 15mins. Supernatant containing released CUT&RUN DNA was recovered and used for Fast DNA extraction by NucleoSpin kit (MACHEREY-NAGEL) as described in the CUT&RUN protocol [63]. CUT&RUN DNA was eluted in 50ul buffer NE and subjected directly to CUT&RUN library preparation.

CUT&RUN DNA libraries were prepared using the KAPA HyperPrep Kit (KK8502, ROCHE) and the KAPA Unique Dual Indexed Adapter Kit (KK8727, ROCHE) following kit protocol (with some modifications in library amplification parameters) in strip PCR tubes. Briefly, DNA fragments after End repair and A-tailing were subjected to adapter ligation reaction with 5μl of 300nM UDI adapters for 16hrs at 8°C in a thermocycler. After bead-based post-ligation cleanup step using KAPA HyperPure Beads (KK8008, ROCHE), library molecules were amplified 14 cycles using parameters described in the CUT&RUN protocol [63]. After bead based post-amplification clean up, amplified DNA library molecules were subjected to double-sided size selection to enrich for adapter ligated DNA fragments in the range of 250–500bp (KAPA HyperPrep Kit protocol Appendix 1.A1). The concentration of amplified and double size cut libraries was measured using Qubit 3 Fluorometer (Invitrogen) and sent to the Genome Sequencing Facility at UT Health San Antonio for Bioanalyzer QC and sequencing. Libraries passing QC were subjected to next generation sequencing on S4 flow cell of an Illumina NovaSeq 6000 sequencer at an average depth of ≥10m reads/sample using 100PE module.

### Immunocytochemistry

Myometrial stem cells grown in hypoxia in a 24 well plate on coverslip coated with attachment factor 1X (GIBCO) were treated with either CCT251545 (100nm) or DMSO for 3.5days. Cells were fixed with 4% paraformaldehyde for 10mins/RT, permeabilized with 0.2% Triton X100 in 2mg/ml BSA on ice for 10mins, blocked with 3% BSA in PBS for 30min/RT followed by incubation with primary antibodies in 1% BSA overnight at 4°C. Cells were then gently washed 3X with PBS, incubated with secondary antibodies at 37°C/30mins in dark, washed 3X again with PBS, and finally incubated with DAPI (1:1000 in PBS) (Sigma-Aldrich) for 10min in dark for nuclear staining. Lastly, cells were washed 3X with PBS, mounted in glass slides by inverting the coverslip in a drop of mounting media (Vector Laboratories) followed by immunofluorescent imaging in a single focal plane at 40x magnification using ZEISS confocal imaging system. Image analysis and quantification was done using ImageJ2 v2.14.

### RT-qPCR

MM SCs grown in hypoxia and treated either with 100nm CCT251545 or DMSO for 4.5h and 3.5days were used for RNA extraction using RNeasy mini kit (QIAGEN). Reverse transcription was performed using iScript Select cDNA synthesis kit (BIO-RAD). Quantitative real-time PCR (RT-qPCR) reactions were prepared with the synthesized cDNA, SSO Advance SYBR green PCR mix (BIO-RAD), and gene specific primer pairs (designed using NCBI Primer Blast) and performed using a CFX384 real time thermal cycler (BIO-RAD). h36B4 (RPLP0 or ribosomal protein P0) was used as endogenous control gene. All the primers were synthesized by Sigma, tested to have a dominant peak for dissociation/melting curve at the end of the qPCR program and their sequences are listed in Table S1.

### Bioinformatic Analysis

#### Analysis of RNA-seq data

Data processing was automated using the RNAsik pipeline [64] and UCSC human genome build (hg38) as the reference genome from raw fastq sequencing data. Read count data were further analyzed using R (v3.6.3). Raw count data was first filtered to exclude genes which lack minimum CPM 1 in at least 1 library across all samples. Differential expression analysis was performed using DESeq2 (v1.41.1) to identify differentially expressed genes between treatment and control samples at each timepoint with default parameters (lfcThreshold = 0 and alpha = 0.1). DESeq2 normalized counts were retrieved using the counts function with the normalized parameter. At each timepoint, PCA was performed using the top 500 variable genes of log transformed count data. Genes meeting both an adjusted p-value threshold of less than 0.05 and an absolute log2 fold change greater than 0.58 (corresponding to absolute fold change of 1.5) were considered significantly differentially expressed.

Gene set enrichment analysis (GSEA) was performed using all the expressed genes. The default signal-to-noise metric was used to rank the genes and perform the enrichment using Broad Institute’s GSEA software (v4.3.3) based on the latest gene set databases (v2023.2). Significantly enriched gene sets were identified as those with FDR q value < 0.05. Gene ontology (GO) analyses were performed using the *Enrichr* functional annotation tool [47].

For transcriptome comparison analysis, we considered two publicly available datasets related to uterine fibroids: GSE128229 [38] and GSE226014 [39]. The raw count tables were harmonized and preprocessed with DESeq2. Batch correction across datasets was performed using sva::ComBat_seq (v3.50.0) [65].

#### Analysis of CUT&RUN data

CUT&RUN sequencing data were processed using the Nextflow core CUT&RUN pipeline (v3.2.1, https://doi.org/10.5281/zenodo.5653535), which includes quality control, support for spike-ins, IgG controls, and peak calling. The target and spike-in genomes were hg38 and E. coli K12 (K12-MG1655), respectively; a blacklist of problematic regions curated for CUT&RUN assays [66] was applied; mitochondrial reads were filtered out; and peaks were called using SEACR. Spike-in normalization occurs prior to peak calling. In short, a scale factor is calculated for each sample by dividing a constant (10,000) by the total number of reads aligned to the spike-in genome. The calculated scale factor is then applied during the conversion of BAM files to BedGraph format, normalizing the read coverage based on spike-in read counts. Differential enrichment of peaks was determined using DiffBind (v3.12.0). DiffBind first generates a consensus peak set by merging peaks present in at least one sample. For each region in this consensus set, DiffBind then calculates affinity scores for each sample by quantifying the read counts within that region, regardless of whether the region was originally called as a peak in that specific sample. Consensus peaks that overlap a blacklisted or DiffBind greylists regions were excluded. Affinity scores were normalized using the default normalization method. A peak was identified as differential if the absolute log2 fold change of normalized affinity scores between Treatment and Control samples was greater than 0.58. Peak annotation was performed using ChIPseeker (v.1.38.0). For super-enhancer calling, rank ordering of super-enhancers (ROSE) was performed using ROSE v1.3.2.

#### Statistics

Applied statistics are mentioned in the corresponding figure legends and software used for data analysis are clarified under Experimental Procedures. *P ≤ 0.05, **P ≤ 0.01, ***P ≤ 0.001. Statistical analysis was performed with GraphPad Prism10, or Microsoft Excel v16.5. For RNA sequencing, three independent replicates of control and CCT251545-treated MM SCs (n = 3) were used, while CUT&RUN analysis was performed with single batch of control and treated MM SCs (n = 1). Immunocytochemistry, immunoblots, and RT-qPCR used three independent replicates of samples.

## Supplementary Material

Supplement 1 **Figure S1. Isolation and characterization of MM SCs. A.** Workflow for processing hysterectomized myometrium for MM SC isolation using the side population (SP) method. **B.** Sorting of MM SP cells by Hoechst 33342 staining. Addition of Hoechst efflux pump ABCG2 inhibitor (Reserpine) eliminates SP population cells. Re-sorting of SP (blue) and non-SP (NSP; green) cells correctly positions them according to their original gating windows. **C.** RT-qPCR was used to quantify mRNA expression levels of nuclear hormone receptor [estrogen receptor α (ESR1), progesterone receptor (PGR)], smooth muscle [calponin 1 (CNN1), α smooth muscle actin (αSMA; aka ATCA2], and stem cell (OCT4, NANOG, SOX2, SUSD2, ABCG2) markers in myometrial NSP cells (blue bars) and SP cells (orange bars). mRNA expression levels were normalized to that of RPLP0 and expressed relative to their levels in MM NSP cells. Data are mean ± SEM of 3 experiments performed in triplicate. Asterisks denote significant differences (*P ≤ 0.05, **P ≤ 0.01; two-sided unpaired t-test in Excel v16.5).
**Figure S2. Validation of Mediator kinase inhibition in MM SCs by CCT251545 treatment. A** and **B.** Representative immunoblot (**A**) and dose response plot (**B**) derived from three independent immunoblots showing impact of increasing CCT251545 concentration on INFγ-stimulated STAT1 serine 727 phosphorylation (pSTAT1^SER727^), a validated Mediator kinase target [28, 30]. MM SCs cells were treated with control DMSO or CCT251545 (4pM-4μM final concentration) for 24hr along with γ-interferon (10ng/ml) for 45 minutes prior to cell harvest and processing of whole cell lysates for immunoblot analysis using the indicated antibodies specific for pSTAT1^SER727^, bulk STAT1, and β-Actin, the latter of which served as an internal loading control. The level of pSTAT1^SER727^ under each condition was determined by quantification of immunoblot signals and normalization of pSTAT1^SER727^ signals to both bulk STAT1 and β-Actin. Statistical analysis was calculated using One-Way ANOVA followed by Tukey’s post hoc test. ****p < 0.0001 compared to INF-γ alone. **C.** Replicate cultures of MM SCs used for RNA-seq analysis in Figs. 1 and 2 were processed in parallel for CDK8/19 inhibition by monitoring INFγ-stimulated pSTAT1^SER727^. MM SP cells were treated with control DMSO or CCT251545 (100nM) for 4.5hr or 3.5d as indicated along with γ-interferon (10ng/ml) for 45 minutes prior to cell harvest and processing of whole cell lysates for immunoblot analysis using antibodies specific for pSTAT1^SER727^, bulk STAT1, and β-ACTIN, the latter of which served as an internal loading control.
**Figure S3. DEGs in MM SCs following 4.5h and 3.5d treatment with CCT251545 show high degree of directional concordance. A.** Venn diagram depicting overlap between DEGs in 4.5h and 3.5d CCT251545 (CCT)-treated MM SCs. Among 167 DEGs identified at 4.5h post-CCT treatment, 104 (~ 62%) were common with DEGs at 3.5d post-CCT treatment, producing a highly significant hypergeometric P value. **B.** and **C.** Overlap of upregulated (**B**) and downregulated (**C**) DEGs from both timepoints shows a high degree of concordance in the direction of change. A more significant concordance among upregulated DEGs is evident from hypergeometric P values above each diagram.
**Figure S4. Principle component analysis (PCA) reveals that Mediator kinase inhibition in MM SCs effectively models established MED12 UF driver mutations. A.** RNA-seq data from MED12 mutant tumors and their paired myometrial tissues (GSE128229 [38]), engineered MED12 G44N UtSMC clones (GSE226014 [39]), and Mediator kinase inhibited MM SCs (this study) were subjected to batch correction using ComBat-seq [65]. Batch corrected transcriptomic profiles were subjected to PCA. Note that the PC1 separates transcriptomes from tissues and cells. **B.** PC2 and PC3 combined to account for 20% of variance and segregate control (DMSO) and CCT251545-treated MM SCs in a manner where the former is placed closer to myometrial tissues (MYO) and latter to MED12 mutant fibroid tumors (LEIO).
**Figure S5. Model for aberrant differentiation of Mediator kinase-inhibited MM SCs.** CDK8 (and/or CDK19) is a general barrier to MM SC differentiation. During normal smooth muscle differentiation, this CDK8-imposed barrier is removed in a tightly regulated manner by physiologic inductive signals, permitting programmed differentiation of MM SCs into uterine smooth muscle cells (top arm). Spontaneous disruption of CDK8 activity (by chemical inhibition or somatic MED12 mutation) triggers unprogrammed MM SC differentiation down an altered path toward tumor initiating cells (TICs) with molecular characteristics of UFs, including extracellular matrix (ECM) production and oncogenic growth (bottom arm). The altered phenotype of CDK8-inhibited MM SCs could derive from enhanced plasticity, permitting these cells to acquire mixed features of myogenic differentiation and other mesenchymal-determined fates. Accordingly, diminished CDK8 activity by physiological signaling maintains myometrial tissue integrity, while spontaneous CDK8 inhibition by genetic or pharmacologic means can be tumorigenic.

## Data Availability

The datasets generated during and/or analyzed during the current study are available in the GEO repository with accession numbers GSE276458 and GSE276459.

## Key messages

- Mediator kinase inhibition in myometrial stem cells (MM SCs) induces spontaneous differentiation.
- Transcriptional changes upon Mediator kinase inhibition recapitulate those of MED12 mutant uterine fibroids (UFs).
- Such transcriptional changes are partially mediated by super enhancer reprogramming.
- Mediator kinase functions to enforce cell states and it’s loss induces cellular plasticity.
