# Supplementary material for: Mediator kinase inhibition drives myometrial stem cell differentiation and the uterine fibroid phenotype through super-enhancer reprogramming
Source: Res Sq. 2024 Dec 16:rs.3.rs-5125876. Preprint. [Version 1] doi: 10.21203/rs.3.rs-5125876/v1 (PMC11702794; doi:10.21203/rs.3.rs-5125876/v1)
Supplement: Supplement 1 — Figure S1. Isolation and characterization of MM SCs. A. Workflow for processing hysterectomized myometrium for MM SC isolation using the side population (SP) method. B. Sorting of MM SP cells by Hoechst 33342 staining. Addition of Hoechst efflux pump ABCG2 inhibitor (Reserpine) eliminates SP population cells. Re-sorting of SP (blue) and non-SP (NSP; green) cells correctly positions them according to their original gating windows. C. RT-qPCR was used to quantify mRNA expression levels of nuclear hormone receptor [estrogen receptor α (ESR1), progesterone receptor (PGR)], smooth muscle [calponin 1 (CNN1), α smooth muscle actin (αSMA; aka ATCA2], and stem cell (OCT4, NANOG, SOX2, SUSD2, ABCG2) markers in myometrial NSP cells (blue bars) and SP cells (orange bars). mRNA expression levels were normalized to that of RPLP0 and expressed relative to their levels in MM NSP cells. Data are mean ± SEM of 3 experiments performed in triplicate. Asterisks denote significant differences (*P ≤ 0.05, **P ≤ 0.01; two-sided unpaired t-test in Excel v16.5). Figure S2. Validation of Mediator kinase inhibition in MM SCs by CCT251545 treatment. A and B. Representative immunoblot (A) and dose response plot (B) derived from three independent immunoblots showing impact of increasing CCT251545 concentration on INFγ-stimulated STAT1 serine 727 phosphorylation (pSTAT1SER727), a validated Mediator kinase target [28, 30]. MM SCs cells were treated with control DMSO or CCT251545 (4pM-4μM final concentration) for 24hr along with γ-interferon (10ng/ml) for 45 minutes prior to cell harvest and processing of whole cell lysates for immunoblot analysis using the indicated antibodies specific for pSTAT1SER727, bulk STAT1, and β-Actin, the latter of which served as an internal loading control. The level of pSTAT1SER727 under each condition was determined by quantification of immunoblot signals and normalization of pSTAT1SER727 signals to both bulk STAT1 and β-Actin. Statistical analysis was calculated usin [file NIHPPRS5125876v1-supplement-1.pdf]

Supplementary Files

This is a list of supplementary files associated with this preprint. Click to download.

- SupplementaryTables.docx
- SupplementaryFigureslegends.docx
- KhadkaJMMSuppFigsRev120924.docx
- CUTRUNroseresults.xlsx
- RNAseqdeseq2results.xlsx
